# A Farewell to Homo Sacer? Sovereign Power and Bare Life in Agamben’s Coronavirus Commentary

**DOI:** 10.1007/s10978-021-09314-x

**Published:** 2021-11-26

**Authors:** Sergei Prozorov

**Affiliations:** Department of Social Sciences and Philosophy, University of Jyväskylä, 40014 Jyväskylä, Finland

**Keywords:** Giorgio Agamben, Sovereignty, Biopolitics, State of exception, Pandemic, Covid-19

## Abstract

The article addresses Giorgio Agamben’s critical commentary on the global governance of the Covid-19 pandemic as a paradigm of his political thought. While Agamben’s comments have been criticized as exaggerated and conspiratorial, they arise from the conceptual constellation that he has developed starting from the first volume of his *Homo Sacer* series. At the centre of this constellation is the relation between the concepts of sovereign power and bare life, whose articulation in the figure of *homo sacer* Agamben traces from the Antiquity to the present. We shall demonstrate that any such articulation is impossible due to the belonging of these concepts to different planes, respectively empirical and transcendental, which Agamben brings together in a problematic fashion. His account of the sovereign state of exception collapses a plurality of empirical states of exception into a zone of indistinction between different exceptional states and the normal state and then elevates this very indistinction to the transcendental condition of intelligibility of politics as such. Conversely, the notion of bare life, originally posited as the transcendental condition of possibility of positive forms of life, is recast as an empirical figure, whose sole form is the absence of form. We conclude that this problematic articulation should be abandoned for a theory that rather highlights the non-relation between sovereign power and bare life, which conditions the possibility of resistance and transformation that remains obscure in Agamben’s thought.

## Introduction

The 2020 coronavirus pandemic quickly became the object of commentary by philosophers and social theorists. Giorgio Agamben has offered highly controversial comments on the subject in a series of short vignettes at the Quodlibet blog, most of which were subsequently collected into a book, entitled *Where Are We Now? The Epidemic as Politics* (Agamben 2021). Agamben is best known for his theory of biopolitics, developed in the multi-volume *Homo Sacer* series, which traced the subjection of life to sovereign power in the Western political tradition from the antiquity onwards. It is therefore hardly surprising that Agamben would address the Covid-19 pandemic as the paradigmatic case of biopolitical governance, which in the argument of his theory proceeds through the inclusive exclusion of bare life in the state of exception, where it ends up exposed to the pure force of the law (Agamben 1998, 2005). Yet, most of his readers were hardly prepared for the virulence of his criticism of the governmental efforts to stop the spread of the pandemic.

Agamben began by questioning the very existence of the pandemic, downplaying the seriousness of the coronavirus infection and presenting it as a pretext for the expansion and the intensification of the state of exception: ‘Once terrorism ceased to exist as a cause for measures of exception, the invention of an epidemic offers the ideal pretext for widening them beyond all known limits’ (Agamben 2021, p. 13). In a subsequent text ‘Clarifications’, Agamben proceeded to infer from the alleged panic about the virus the fact that ‘[our] society no longer believes in nothing more than bare life. It is now obvious that Italians are ready to sacrifice practically everything—their life conditions, their social relationships, their work, even their friendships, as well as their religious and political convictions—to the danger of getting sick’ (2021, p. 17).

This willingness of the society to suspend its interactions and relationships for the sake of survival enables the proliferation of exceptional governmental interventions that make the state of exception the ‘new normal’: ‘[People] have been so used to living in conditions of perennial crisis and emergency that they seem not to realize that their life has been reduced to a purely biological state. A society that exists in a constant state of emergency cannot be free’ (2021, p. 18).

In later comments Agamben continued with this dual line of criticism, addressing various ways, in which the fear of the coronavirus reduces our social lives to the preoccupation with bare life, which in turn legitimizes the emergency measures undertaken by governments. He also issued damning invectives against the church, which allegedly betrayed its mission ‘to keep watch over human dignity’, the lawyers who remained silent in the face of the proliferation of states of exception, and, finally, medicine itself, which he accused of having become a new religion (Agamben 2021, p. 34–37; p. 49–54).

Agamben’s invectives have been received critically by numerous interlocutors, including such fellow philosophers as Jean-Luc Nancy (2020) and Roberto Esposito (2020). Critics have noted the uncanny proximity of Agamben’s position to that of Covid denialists or skeptics, from Bolsonaro to Trump, and questioned the conspiratorial tone of his claims about the ‘invention’ of the epidemic (see Christiaens 2020; van den Berge 2020). While we are largely in agreement with this line of criticism, our objective in this article goes beyond the adjudication of Agamben’s writings on the coronavirus as to their veracity or political correctness. We are more interested in analyzing these writings in order to illuminate some fundamental issues with Agamben’s political thought that might have otherwise remained obscure.

It is important to stress that we do not intend to infer from Agamben’s opinion pieces a disqualification of Agamben’s philosophical approach, as if a philosopher must be correct in their every opinion for their philosophy to merit any consideration. And yet, it would also be incorrect to try to rigorously separate these opinion pieces from Agamben’s political philosophy, if only because in the former he applies and refines the concepts developed in the latter. While *Where Are We Now?* might understandably lack the methodological rigour of Agamben’s more academic works, it nonetheless offers a powerful exemplar or *paradigm* of Agamben’s reasoning and is therefore a legitimate objects of critical analysis.

The focus of this article is on the relation between two fundamental concepts in Agamben’s political thought that he continued to rely on in his coronavirus writings: *sovereign power*, exercised in the form of the state of exception, and *bare life* included in this state only as negated or excluded. His claims above about the pandemic reducing our social life to bare life and enabling governance by exceptional measures are not merely empirical observations that could be refuted by pointing to contrary examples—e.g. of altruist and solidarist behaviours observable throughout the pandemic, or the hurried lifting of many of the restrictions that, Agamben argued, would be maintained even after the pandemic ends. These claims rather recall much earlier theoretical theses about the relation of sovereign power to life that date back to Agamben’s articles from the 1990s and the first volume of the *Homo Sacer* series that preceded and, some might say, prophesied the rise of exceptional or emergency governance measures worldwide (Agamben 1998, 2000).

Similarly, the response to Agamben’s coronavirus statements as overly exaggerated only brings to mind a similarly incredulous reception of the claims in *Homo Sacer* about us all being ‘virtually *homines sacri*’ and the camp as the ‘nomos of the modern’ (Agamben 1998, p. 155; 166), which led to Agamben being accused of ‘jarringly disconcerting claims’ (La Capra 2007, p. 133), ‘wild statements’ (Laclau 2007, p. 22) and ‘unregulated decisions’ (Norris 2005, p. 273). It would thus be incorrect to read Agamben’s coronavirus statements as polemical exaggerations at odds with the measured tone of his more academic writings. Polemical exaggerations were abundant in the latter writings as well, which permits us to raise the question of whether this tendency towards hyperbole and exaggeration might not be an individual idiosyncrasy, but instead arises from the conceptual logic at work in Agamben’s political theory, particularly the relation between sovereign power and bare life. In this article we shall therefore venture a *critical reconstruction* of this logic, drawing on both Agamben’s theoretical texts and his coronavirus commentary, in order to point out its problematic features and outline a pathway of the resolution of these problems from within the framework of the theory itself. We shall neither attempt to insulate Agamben’s political theory from his incidental commentary on current affairs nor infer from the problematic nature of this commentary the need to discard this theory as a whole, but rather use this commentary as an occasion for immanent criticism of the central tenets of the theory. Thus, while we shall argue that Agamben’s articulation of the relation of sovereign power and bare life in *Homo Sacer 1* is fundamentally flawed, we shall also demonstrate how these flaws may be remedied with the help of Agamben’s own theoretical logic, particularly the idea of non-relation, which first appeared in *Homo Sacer 1* but is most fully elaborated in *The Use of Bodies*, the final volume of the *Homo Sacer* series.

*Homo Sacer* famously begins with a forceful assertion of the relation between sovereign power and bare life: ‘The inclusion of bare life in the political realm constitutes the original—if concealed—nucleus of sovereign power. It can even be said that the production of a biopolitical body is the original activity of sovereign power’ (Agamben 1998, p. 6). By suspending the law in the state of exception, sovereign power is able to access and capture not merely some particular form of life but life as such. The entire trajectory of Western politics consists in the encounter of power devoid of all normative content with life devoid of its form. This is why for Agamben the paradigmatic space of modern politics, in which this trajectory culminates, is the camp: ‘Insofar as its inhabitants were stripped of every political status and wholly reduced to bare life, the camp was also the most absolute biopolitical space ever to have been realized, in which power confronts nothing but pure life, without any mediation.’ (1998, pp. 170–171)

This conceptual structure forms the basis of Agamben’s critical response to the governance of the coronavirus pandemic. The pandemic is problematic insofar as it brings sovereign power and bare life ever closer together. On the one hand, the introduction of the state of emergency in Italy and many other European countries has reduced the exercise of power to its pure form of sovereignty, revealing the dependence of the law on its apparent opposite:

We have been accustomed for some time to the ill-advised use of emergency degrees through which executive power effectively replaces legislative power, abolishing the separation-of-powers principle that democracy is defined by. In this case, however, all limits have been overstepped: it seems that the words pronounced by the Prime Minister and by the head of the Civil Protection Department have the immediate validity of law (as was once said of the words of the *Führer*. (Agamben 2021, p. 36)

On the other hand, the ascent of the coronavirus to the top of the political agenda has reduced political life to mere ‘survival’ (Agamben 1999, pp. 132–135), as good life has been sacrificed for the protection of the bare life that sustains it. ‘[Bare] life, and the fear of losing it, is not something that unites people; rather, it blinds and separates them. What are human relationships becoming in a country that has resigned itself to the idea of living like this for the foreseeable future? And what is a society that values nothing more than survival?’ (Agamben 2021, p. 18).

It is as if Agamben’s theory, initially developed in the aftermath of the end of the Cold War, has finally been fully vindicated: what we observe globally is the reduction of politics to exceptional sovereign government of life stripped of its form and reduced to bare survival. From this perspective, even relatively innocuous measures, from compulsory face masks in public places to the shift to online education, exemplify the far more ominous tendency towards the articulation of the law devoid of content with the life devoid of form, whose paradigmatic site is the camp and whose exemplary figure is *homo sacer*. This is why Agamben is able to argue that a ‘country that decides to renounce its face, to cover with masks the faces of its citizens everywhere’ has ‘purged itself of any political dimension’ (2021, p. 87) or that ‘[professors] who agree to subject themselves to the new online dictatorship and to hold all their classes remotely are the exact equivalent of those university professors who, in 1931, swore allegiance to the Fascist regime’ (2001, p. 74).

Rather than simply dismiss these admittedly preposterous statements as exaggerations, in this article we shall pose the question of whether the conceptual logic at work in them can be sustained as such, even in a more moderate version. We shall argue that the articulation of sovereign power and bare life, first posited in *Homo Sacer 1* and reaffirmed in *Where Are We Now?*, is problematic due to the belonging of the two concepts to different planes, respectively empirical and transcendental. It is only by extending their field of application beyond these planes that Agamben is able to conceive of their articulation. In the following section we shall demonstrate how his account of the sovereign state of exception collapses a plurality of empirical states of emergency into a zone of indistinction between different exceptional states and the normal state and then elevates this very indistinction to the transcendental condition of intelligibility of politics as such. In the third section we shall analyse the inverse move of recasting bare life, originally posited as the transcendental condition of possibility of positive forms of life, as an empirical figure, whose sole form is the absence of form. In the final section we shall argue that the resulting articulation of the empirical and the transcendental, whose concrete exemplar is *homo sacer*, is fundamentally flawed insofar as it establishes as the inescapable horizon of Western politics the relation that appears highly dubious and contrived. While this criticism does target the central tenet of the first volume of *Homo Sacer*, it does not extend to the entirety of the *Homo Sacer* series or Agamben’s wider project, which instead provides us with conceptual resources for an alternative approach that would highlight *non-relation* between sovereign power and bare life—a notion that has remained rather obscure in Agamben’s thought (see McLoughlin 2009).

## Sovereign Power: From the Empirical to the Transcendental

In *Homo Sacer* Agamben defines the ‘logic of sovereignty’ in Schmittian terms as the decision on the exception (Agamben 1998, pp: 15–29). Sovereign power is realized in the state of exception and the state of exception is the *topos* of sovereign power. It is only by means of suspending itself in the exception that law can get access to life. By suspending its content and revealing itself as pure force, legal norm becomes indistinct from fact and can then graft itself onto life in its sheer facticity, stripping it of all its positive forms. In *Homo Sacer* and *State of Exception* Agamben uses the notion of state of exception in two related yet importantly different senses. On the one hand, he follows Schmitt’s understanding of the decision on the exception as a sovereign act par excellence. The Schmittian state of exception is by definition *distinct* from a normal state and derives its very force from the rupture with the normal state: ‘In the exception the power of real life breaks through the crust of a mechanism that has become torpid by repetition’ (Schmitt 1985, p. 15).

On the other hand, Agamben repeatedly highlights the tendency that was not explicitly addressed by Schmitt, even as it certainly characterized the period in which he was writing, i.e. the *expansion* of the state of exception to engulf the normal state itself, so that exception becomes the norm and thereby is *no longer distinct* from it. Contemporary examples of this indistinction are numerous, ranging from the expansion of administrative regulation that sidelines parliamentary procedures to wars and military operations undertaken in blatant disregard of international law. ‘The normative aspect of law can thus be obliterated and contradicted with impunity by a government violence that—while ignoring international law externally and producing a permanent state of exception internally—nevertheless still claims to be applying the law’ (Agamben 2005, pp. 85–86).

The state of exception is thus at once a violent act of breaking with the norm, which defines sovereign power, and the new norm itself, insofar as such ruptures have become ever more frequent and, as it were, regular. This is why Agamben is able to argue that the states of emergency declared in Italy and elsewhere during the pandemic were at once something radically new and merely yet another illustration of that to which we have already become accustomed. ‘[What] the epidemic is making clear is that the state of exception, which governments have *for quite some time* accustomed us to, has finally become the norm. More serious epidemics have happened in the past, but *nobody ever dared* declare for that reason a state of emergency, which keeps us from moving like the present one does’ (Agamben 2021, p. 18. Emphasis added). The conjunction of the old and the new, the familiar and the unprecedented, is a well-known argumentative strategy in Agamben’s work and was criticized by, among others, Jacques Derrida:

Agamben, giving nothing up, like the unconscious, wants to be twice first, the first to see and announce, and the first to remind: he wants both to be the first to announce an unprecedented and new thing, what he calls this ‘decisive event of modernity’ [the birth of biopolitics], and also to be the first to recall that in fact it’s always been like this, from time immemorial. He is the first to tell us two things in one: it’s just happened for the first time, you ain’t seen nothing yet, but nor have you seen, I’m telling you for the first time, that it dates from year zero. (Derrida 2009, p. 330)

The state of exception perpetually *keeps becoming* the rule, every time as if for the first time, so that it brings to clarity what has been happening ‘for some time’ but only by doing something ‘no one ever’ had thought of doing. Thus, the resort of governments worldwide to the declarations of temporary states of emergency in the first wave of the pandemic in Spring 2020 is something at once unprecedented and unsurprising. There nonetheless remains a question of why these states of emergency needed to be declared *at all*, if, as Agamben has long argued, exceptional measures have long been in place already. The resort of democratic governments to legislation on the state of exception or emergency early on in the pandemic appears to throw doubt on Agamben’s argument about the creeping expansion of the state of exception, as despite all the paralegal regulations and administrative decrees that have apparently side-lined parliamentary law-making, the states of exception during the pandemic were explicitly authorized by national parliaments without either contradicting or sidelining the principles of democratic governance.

Agamben’s approach ventures to disable this objection from the outset by invoking yet another indistinction, this time between democracy and totalitarianism. Agamben famously opened *Homo Sacer 1* with a still controversial claim about the ‘inner solidarity of democracy and totalitarianism’ (Agamben 1998, p. 10), which is grounded precisely in their shared biopolitical orientation.

It is almost as if, starting from a certain point, every decisive political event were double-sided: the spaces, the liberties and the rights won by individuals in their conflicts with central powers always simultaneously prepared a tacit but increasing inscription of individuals’ lives within the state order. (Agamben 1998, pp. 121–122)

However, the experience of twentieth century totalitarianism differs so significantly from the experience of Western democracies that the argument about their indistinction appears barely defensible (cf. Rasch 2007). In terms of the two senses of the state of exception (as a rupture with the norm and as the ‘new normal’), totalitarian regimes differed from Western democracies in no longer requiring to declare the first state of exception because, *unlike the latter*, they had actually realized the second (see Mesnard 2004). In totalitarian regimes the sovereign power of exception is indeed realized *normally*—i.e. without any need for the state of exception to be *declared* in the Schmittian eruption of the power of real life against the torpid legal ‘mechanism’ (cf. Agamben 1998, pp. 170–171). In contrast, in liberal democracies, whatever their faults, the suspension of legal norms requires the declaration of the state of exception, according to precise legal procedures and usually for a limited period of time. Yet, Agamben’s approach cannot register this or, for that matter, any other distinction between the ways in which the state of exception functions in different regimes. This is because, as we recall, the ‘production of the biopolitical body’ (i.e. bare life caught in the state of exception) is the ‘original activity of sovereign power’ (Agamben 1998, p. 6). All historical instances of the state of exception, from the Roman Empire via Nazi Germany to the pandemic-stricken Europe of our time, merely actualize this ‘original activity’.

Thus, Agamben’s favourite trope of indistinction is applied *twice*: firstly, all instances of the state of exception are indistinct as actualizations of the original activity of sovereign power and, secondly, the state of exception is indistinct from any putative normal state due to its sheer regularity that makes it appear as ‘torpid’ as the crust of the legal mechanism that Schmitt’s decision broke through. By means of these two indistinctions the state of exception loses all empirical intelligibility, as it becomes the condition of our access to politics as such, thereby retreating into the transcendental realm. As a result of this retreat, we are able to recognize what politics is but only at the price of recognizing it as *always the same*: the indistinction between sovereign and bio-power is realized indistinctly in democratic and totalitarian regimes so that the exception is indistinct from the norm, and so on.

Why does this retreat into the transcendental take place? It appears that Agamben falls victim to Schmitt’s theory (in the same way as Schmitt, according to Leo Strauss (1976, p. 103), fell victim to liberalism) by adopting his logic of argumentation with a *minus sign* preceding it. The state of exception was in Schmitt the *high point* of sovereign power, the point where law would merge with life, rupturing the crusty mechanisms of the system. For Agamben, the state of exception is a particularly *low point*, where life is stripped of its form and caught up in the ban. Yet, this formal negation does nothing to question Schmitt’s argument that politics is ‘all about’ the exception and that the measure of sovereignty is the capacity to *decide* on the exception that does not pre-exist it (see List 2020). As a result, the decision on exception becomes generalized across the most diverse political regimes as something that precedes and exceeds them, something always already constitutive of the very norm it suspends. As long as there are empirical examples of both democratic and totalitarian regimes relying on such decisions, their ‘inner solidarity’ is apparently always already proven.

A side effect of this inverted Schmittianism is the almost omnipotent efficacy granted to the sovereign decision in Agamben’s approach, which contrasts strongly and unfavourably with the rather more nuanced approach of the author that has otherwise been a major influence on Agamben, i.e. Walter Benjamin. In his *Origin of German Tragic Drama* Benjamin presented a historical example of *baroque sovereignty,* in which the sovereign was not at the height of its powers when declaring the state of exception but was rather caught up in the exception that was *always already underway*, and ‘the most important function of the prince [was] to exclude this’ (2003, p. 55). What is important about this approach is the figuration of the exception in decidedly un-Schmittian terms as a condition that demands the sovereign’s response, rather than a declarative decision—a response that is difficult and, more often than not, bound to fail. ‘The sovereign, who is responsible for making the decision on the state of exception, reveals, at the first opportunity, that it is almost impossible for him to decide’ (Benjamin 2003, p. 71).

We need only recall Benjamin’s series of the figures of tyrant, martyr and intrigant that populate the baroque political space (Benjamin 2003, pp. 70–88). While the tyrant and the martyr exemplify the failed response to the challenge of the exception, which is why the former so often becomes the latter, the intrigant succeeds (and survives) by giving up on the solemn symbolism of sovereignty, operating instead in the terrain, already modified by the exception and seeking nothing more than to *manipulate* it to its own advantage. When Benjamin later speaks of a ‘real state of exception’ in the eighth of his ‘Theses on the Philosophy of History’ (1968, p. 263), he arguably has in mind precisely this state, which is not a product of sovereign power, as Schmitt’s *fictive* states of exception all are, but exists independently of it and demands a decision that is difficult and risky.

This reading of the exception seems to us to be rather more appropriate for the coronavirus crisis and other complex emergencies of our time than their interpretation as a conspiracy of state power to realize a state of exception in every aspect of our existence (see List 2020). Epidemics, heat waves, forest fires, floods, hunger, and climate change that causes most of the above exist as emergencies that may or may not become the justification for states of exception, which, more often than not, reflect not the splendour of sovereign decisionism, but the trembling before the decision that might be entirely ineffective, come too late or otherwise end in failure. Given the initial and even ongoing uncertainty and lack of knowledge regarding the origins and effects of the coronavirus, the states of exception introduced by governments worldwide can hardly appear as signs of their omnipotence but rather reflect their impotence in the face of the situation that is genuinely exceptional, not as a result of any sovereign decision but largely irrespective of it.

While Agamben certainly sides with Benjamin in his analysis of the esoteric debate between Benjamin and Schmitt in *State of Exception*, he ultimately subsumes his insights under the Schmittian concept, so that even chaos and anomie end up reinscribed within the juridical order (Agamben 2005, pp. 56–57; see List 2020). This reinscription obscures arguably the most important insight of Benjamin’s analysis: not only is it possible to conceive of an exception that *challenges* rather than fortifies sovereign power, but it is also possible to *oppose* sovereign power by bringing about a state of exception, in which the latter would not be able to decide on anything whatsoever. It is no longer possible to define sovereign power in terms of its production of bare life through the state of exception, if only because the sovereign may easily find itself reduced to bare life in the exception that is *not of its making*.

Instead, practices of sovereignty unfold in the continuum, whose extreme points are the Schmittian state of exception effectively enacted by the sovereign and the Benjaminian state of exception, in which the sovereign’s inability to decide leads it into ruin. Along this continuum we could locate numerous intermediate possibilities, in which the exception is neither merely a product of sovereign will nor an objectively given situation, but a certain combination of both. By the same token, along this continuum we would be able to locate different kinds of political regimes, in which the state of exception would be realized differently: e.g. a spatio-temporally circumscribed state of emergency instituted by parliamentary vote in response to a specific emergency, as opposed to an indefinite suspension of fundamental civil rights and liberties. Instead of Agamben’s general indistinction we would rather be able to make *numerous* distinctions between the ways, in which different states, regimes or forms of government have dealt with the challenge of the exception.

In contrast, the only way to sustain the thesis of indistinction is to insulate it from any empirical consideration by recasting it in the inverse-Schmittian manner as the quasi-transcendental condition of possibility of any politics whatsoever, be it sovereign or bio-politics, democratic or totalitarian, ancient or modern, etc. By virtue of such recasting, Agamben’s persistent comparisons of contemporary liberal democracies with fascist and Nazi regimes are protected from any empirical refutation: ‘the production of a biopolitical body is the original activity of sovereign power’, hence any empirically observable distinctions between these states of exception must be, as it were, distinct manifestations of what is originally indistinct (cf. Kalyvas 2005, pp. 111–12).

Our argument might recall the frequent criticism of Agamben’s ‘ontologization’ of sovereignty and biopower, which has been advanced repeatedly since the publication of *Homo Sacer 1* (see Passavant 2007; Toscano 2011). It is indeed true that Agamben models his political theory as a correlate of ontology: ‘Today *bios* lies in *zoe* exactly as essence, in the Heideggerian definition of Dasein, lies in existence’ (Agamben 1998, p. 188). Yet, the problem we are addressing here is distinct from this kind of ‘ontologization’. What is at issue is not that the question of power is relocated to the level of being, or, rather, the difference between being and beings, but rather that a plurality of historical forms of power is first collapsed into a ‘zone of indistinction’, which is then elevated to the condition of our access to the political as such. It is not a matter of opposing the ontological to the historical, if only because the two can be brought into alignment in either the Heideggerian or the Foucauldian manner, but rather of inferring from the indifferent series of empirical forms the transcendental condition of their sheer accessibility.

While this retreat into the quasi-transcendental might be an unorthodox and audacious move on Agamben’s part, there remains a question about its utility. To diagnose the present or any other state of affairs as a global state of exception, itself indistinct from the normal state and hence not really exceptional, is hardly a valuable insight, even when it might be accepted as a logical implication of the definition of the ‘original activity’ of sovereign power. Yet, if Agamben’s diagnoses are correct only ‘by definition’, what is then the value of this definition itself, if all that it can do is level all empirically observable distinctions and subsume the most diverse phenomena under a very general category that offers little or no orientation in the plurality of emergencies and exceptions that surround us? All that is gained by this transcendental recasting of the logic of sovereignty is the certitude of the oppositional stance, which is spared the need for critical discernment, since all distinctions have now been erased.

In the following section we shall address the counterpart to sovereign power in Agamben’s theory, i.e. the concept of bare life, and argue that with regard to this concept Agamben makes a diametrically opposite move of inferring a series of empirical actualizations from a quasi-transcendental presupposition.

## Bare Life: From the Transcendental to the Empirical

We have seen that it is impossible to pass from empirical states of exception to the state of exception ‘as such’, other than by obliterating all distinctions and ending up with an ultimately vacuous concept of the ‘original activity’ of sovereign power. What about the concept of bare life, which is the object of sovereign power in Agamben’s approach?

In Agamben’s theory of biopolitics, this concept functions as the transition from *zoe* to *bios*: for any positive form of life (*bios*) to be instituted, *zoe* (life as such, devoid of qualifications) must be included into it in the strictly *negative* mode of exclusion: something presupposed and negated at once, presupposed *as* negated. While Agamben’s text occasionally slips into the identification of two terms (Agamben 1998, p. 6), in the logic of his argument bare life does *not* precede politics but is rather its *product*, a result of the inclusion of *zoe* into *bios* that cannot be identical to *zoe* itself. Rather than being natural, bare life is in a sense always *de-natured* as a result of its inclusion into the political order. From this perspective, it would be more appropriate to speak of a ‘bared’ or ‘denuded’ life: the nakedness of this life has no connotations of natural innocence whatsoever but rather presupposes a violent act of stripping off all positive attributes of life (Agamben 2010, pp. 55–91).

This mode of implication of *zoe* in *bios* is exactly the same as the one between *phone* (natural sound) and *logos* (language), addressed in Agamben’s *Language and Death* (1991). In both cases, the first term is implicated in the second as its negative foundation (Kotsko 2020, pp. 75–76).

The question ‘in what way does the living being have language?’ corresponds exactly to the question ‘In what way does bare life dwell in the polis?’ The living being has *logos* by taking away and conserving its own voice in it, even as it dwells in the polis by letting its own bare life be excluded, as an exception, within it. Politics therefore appears as the truly fundamental structure of Western metaphysics, insofar as it occupies the threshold on which the relation between the living being and the logos is realized. There is politics because man is the living being who, in language, separates and opposes himself to his own bare life and, at the same time, maintains himself in relation to that bare life in an inclusive exclusion. (Agamben 1998, p. 8)

In this manner, bare life serves as the negative foundation of all forms of life, produced in positive political orders. While these positive orders are certainly different and produce different forms of life, the condition of possibility of this production is the same for all of them: as we recall, ‘the inclusion of bare life in the political realm constitutes the original—if concealed—nucleus of sovereign power’ (Agamben 1998, p. 6). Yet, from this follows an important consequence: the concept of bare life belongs squarely to the order of transcendental presuppositions and could not possibly have an empirical referent. Simply put, *bare life does not exist*; it is rather what must be presupposed for forms of life (*bioi*) to exist as such. Every form presupposes the formless substrate that must have preceded its formation, yet has always already been negated by it. Thus, any use of the concept as a sociological category, let alone as a journalistic catchword, is problematic and leads to conclusions as vulgar as they are absurd. Every actually existing life is *in a form*, however unappealing or worthless this form might appear to some observers.

This is why Agamben’s presentation of a series of ‘examples’ of bare life at the end of *Homo Sacer 1* is so uncanny: surely, the Fuehrer of the Third Reich and the inmate of the camp dwell in somewhat distinct forms of life, just as the experimental scientist and the comatose patient inhabit conditions that are quite different from each other (Agamben 1998, pp. 182–197). What is then the purpose of bringing them together into yet another series that, as Agamben recognizes, appears ‘extreme if not arbitrary’ (1998, pp. 186–187)? The reason he brings these different figures together is to illustrate the instability, if not outright collapse, of ‘the classical distinction between *zoe* and *bios’* (Agamben 1998, p. 187). Yet, this instability has already been posited a priori as a matter of definition of biopolitics in terms of the inclusive exclusion of *zoe* into *bios,* according to which the ‘classical distinction’ between the two was never stable to begin with (Agamben 1998, pp. 1–8). This is why this series of extreme and arbitrary examples could continue almost indefinitely: there is certainly no shortage of examples of the indistinction between life and its form, which were never distinct in the first place. Yet, crucially, *none* of these examples actually succeed in presenting us with a life *wholly devoid of form*—a genuinely ‘bare life’ remains a presupposition that does not find an empirical manifestation.

Let us consider what is undoubtedly most extreme example of bare life in Agamben’s entire *oeuvre*: the Muselmann of *Remnants of Auschwitz*, the inmate of the Nazi camps from whom all attributes and qualifications have been violently stripped away: ‘[it] is the final biopolitical substance to be isolated in the biological continuum. Beyond the Muselmann lies only the gas chamber’ (Agamben 1999, p. 85). Having described in harrowing detail the way Muselmänner were deprived of all human features, including the potentiality for speaking, Agamben surprisingly concludes by citing the testimony of the former Muselmänner: at least some lives stripped of their form were in fact able to regain it and testify to having been ‘bared’ (1999, pp. 166–171). These witnesses are able to testify to the destruction of their form of life, yet the sheer fact of this testimony demonstrates that this destruction is not complete and irreversible. It is never bare life that speaks the words ‘I was a Muselmann’ (Agamben 1999, p. 165).

Of course, millions of Muselmänner did not survive to testify to their lives being stripped of their form, which again suggests that the Nazi production of the ‘final biopolitical substance’ only succeeded in constructing an extreme moment of ‘survival’ that was followed by life either regaining its form or being extinguished in death (Agamben 1999, pp. 132–133). It is beyond doubt that lives can be subjected to the kinds of suffering that destroy all attributes of humanity. Yet, this damaged life is nonetheless never entirely without form, as long as it continues to exist. In short, even the most extreme and abominable practices of dehumanization fail to attain the empirical correlate of bare life as the transcendental presupposition of the constitution of any form of life whatsoever.

Yet, the example of the Muselmann is also helpful for grasping the problem with applying the concept of bare life empirically to criticize particular societal behaviours and choices, as Agamben does repeatedly in his comments on the coronavirus crisis. Surely, the Italian ‘society [that] no longer believes in nothing more than bare life’ (Agamben 2021, p. 17) does not wish to sacrifice all its social relationships for the life of the Muselmann or even the rather less denuded life of the *homo sacer*? Even if this society ‘values nothing more than survival’ (Agamben 2021, p. 18), this clearly does not mean that this sole value can be represented by the Muselmann, beaten and tortured into utter submission yet still somehow alive. It seems evident that whatever life the Italian and other societies value and believe in is not a bare(d) life produced by sovereign power but a life endowed with a certain form, a form that might not appeal to Agamben due to its superficiality or triviality, but a form nonetheless.

This is why it is somewhat embarrassing to read Agamben’s invectives against the hapless Italians that view their neighbours as possible virus spreaders and obediently tolerate curfews and other restrictions, including even the prohibition on the funerals of the victims of Covid-19. While we may agree with some of these moral judgments and disagree with others, what is problematic is casting them in terms of bare life, which now becomes a criterion in terms of which actual forms of life could be valorized or dismissed. The point is not simply that Agamben’s vitriolic opposition to social distancing and mask-wearing ignores the way these measures both presuppose and effect a much stronger degree of solidarity and mutual assistance than the cavalier and reckless behavior of Covid-dissidents who affirm their ‘freedom’ against governmental regulations. More importantly, Agamben’s decision to frame this opposition in terms of attributing to the behaviours he dislikes the ‘formless’ character of bare life paves a way for a new moralism, just as insipid as the one postulating a particular version of ‘good life’. From now on the philosopher would no longer teach people what a good life is but rather accuse the lives he dislikes of being formless and thus not even properly human (Agamben 2021, p. 58):

[How] did it happen that an entire country has, without even realizing what was happening, collapsed both ethically and politically in the face of an illness? Without our having even noticed, or perhaps, having pretended not to notice, the threshold between humanity and barbarism has been crossed. (Agamben 2021, p. 34)

Our point is not that Agamben’s diagnosis must be inverted and the accusation of barbarism should be reserved for Covid-denialists, anti-maskers and conspiracy theorists, but rather that this mode of reasoning as such has something ludicrous about it. Rather than debate possible deficiencies and disadvantages of particular forms of life, this discourse seeks to accuse the opponent of their life not having a form *at all*, which is a self-defeating enterprise that only succeeds in hurling a contradictory insult: ‘your form of life is formless’.

While, as we have seen, there are too many *historical referents* of the state of exception, which renders problematic all attempts to subsume them under a single concept of sovereign power, bare life presents us with the inverse problem of a concept *without a referent*, functioning as a presupposition that permits positive forms of life to be constituted without itself assuming a constituted form. If it is impossible to pass from numerous historical states of exception to a single concept of the state of exception *as such*, it is just as impossible to find a single empirical referent of bare life: whatever life you take, it is never bare. There is an understandable temptation to dismiss particularly vapid or impoverished forms as somehow deficient and thus deformed or formless. Yet, as a result of giving in to this temptation, bare life loses its transcendental status and enters the empirical field as a designator of whatever form of life we find lacking or deficient. We thereby lose a valuable instrument of the analysis of political constitution of forms of life and gain nothing more than a dubious term of cultural criticism, which, moreover, may easily be weaponized against the critic in question.

As we have seen, Agamben’s use of the concepts of sovereign power and bare life extends their field of operation, thereby enabling their eventual articulation. This move may be the most familiar *signature* of Agamben’s political theory, an operation on signs and concepts that endows them with a historically specific function and intelligibility (Agamben 2009, pp. 33–79). It is this signature that accounts for the often remarked tendency in Agamben’s thought toward hyperbole and exaggeration (Abbott 2014, p. 20; Rasch 2007, p. 100; Laclau 2007, p. 22; Fitzpatrick 2005, p. 55): the blurring of the distinction between the empirical and the transcendental permits him to present conditions of possibility as empirical entities and empirical states of affairs as transcendental origins. Thus, the sovereign state of exception is derived from a series of distinct and diverse historical events, whose collapse into a ‘zone of indistinction’ enables its retreat into the quasi-transcendental plane as the ‘original activity’ of sovereign power. In contrast, bare life, which is first posited as a presupposition for the constitution of any form of life whatsoever, increasingly figures as an empirical attribute, launching a series of ‘extreme if not arbitrary’ examples of lives, whose sole form is presumably the absence of form. The two concepts thereby leave the initial sites of their formulation and move towards each other like *opposite vectors*, meeting halfway and converging in the figure of *homo sacer*, a life stripped of its form and abandoned to the force of law stripped of its content.

If we maintain the separation between empirical and transcendental planes, we end up with a rather different image. If the state of exception always remains empirical and does not enter the quasi-transcendental realm, in which bare life functions as the presupposition of positive forms, the two concepts can never form an articulation, even if one postulates their movement towards each other. The vectors in question might well have opposite directions but, belonging to different planes, they remain *antiparallel* and do not intersect. Sovereign power may ceaselessly *presuppose* bare life but cannot actually *produce* it and remains resigned to regulating a plurality of positive forms of life. By stripping life of its form it can only succeed in demonstrating that this form was there to begin with and some of it may even survive the effort of denuding, much as the Muselmänner who bring their deprivation of language to speech as such. Sovereign power has no access to life as such, because there is no such thing as sovereign power ‘as such’: there are only ever particular forms of power grappling with all kinds of forms of life except for bare life, which cannot itself enter the series of forms that it enables. The conjunction ‘and’ in the subtitle of *Homo Sacer 1*, ‘Sovereign Power and Bare Life’, thus denotes not the articulation of the two concepts but a radical *chasm* that cannot be bridged other than in bad faith, by stretching the field of application of these concepts far beyond the limits of their explanatory power.

## Caesura: From Articulation to Non-Relation

We have demonstrated that in the commentary on the pandemic in *Where Are We Now?* Agamben follows the logic of his earlier theoretical texts in extending the field of the application of the concepts of sovereign power and bare life beyond the original sites of their formulation, so that the two concepts end up meeting halfway and articulated in the figure of *homo sacer*, which thus becomes the paradigmatic figure of Western politics.

Yet, if this articulation is impossible, if the two concepts never meet and remain on different planes, then the figure of *homo sacer* becomes ever more blurred and recedes back into ancient history, whence it was derived. This historical figure could only be posited as a paradigm of the political subject in the Western tradition, when politics itself was defined in terms of the capture of bare life in the state of exception, and its paradigmatic status becomes unsustainable as soon as such a capture is no longer seen as the ‘original activity’ of sovereign power. Instead, we end up with a plurality of states, exceptional or regular, confronting a plurality of forms of life in a variety of ways that do not follow a single logic or actualize any original activity. In none of these confrontations does power attain a hold upon life as such or bare life, which is the only reason why all these forms of power can be and frequently are resisted. This resistance does not consist in obstinately holding onto a particular form or affirming the formlessness of life but retains the potentiality for transformation from within whatever form one happens to dwell in.

It is precisely bare life as a quasi-transcendental presupposition that permits this transformation to be thought and practiced. If positive forms were all there is and they were entirely constructed by sovereign power, then we would be unable to resist capture under these forms, as our lives would be wholly determined by them. It is only because life is transcendentally (and only transcendentally!) bare that no form may assume ontological priority and it is possible to resist every effort of formation or deformation from within the form that one happens to be in. Since bare life is a condition of any possible transformation that itself is entirely inaccessible to it, one is never identical to one’s identity, thereby retaining the potentiality for being otherwise which is entirely distinct from the injunction to become someone or something else (Agamben 2010, pp. 44–45). Thus, while the empirical use of the concept of bare life achieves little more than the escalation of polemic about some lives being ‘poor form’, retaining and reaffirming this concept as a transcendental presupposition permits us to identify the conditions of possibility of resistance and transformation that many have found lacking in Agamben’s work (Bernstein 2004; Patton 2007; Whyte 2009).

Our critical reconstruction of Agamben’s argument may encounter an objection, insofar as it is *itself* based on a key distinction between the empirical and the transcendental, while Agamben’s approach is based on the assumption of the blurring and erasure of all distinctions as the ‘original activity’ of sovereign power. Thus, while we have argued for the impossibility of the articulation of the state of exception and bare life due to the chasm between the planes these concepts occupy, Agamben or his followers might counter that any such gap have always already been occluded, enabling the articulation of the empirical state of exception and transcendental bare life. The concrete embodiment of this articulation, *homo sacer*, is then precisely the ‘empirico-transcendental doublet’, whom Foucault famously posited as the key figure of the modern episteme, at once the condition of possibility of all knowledge and the empirical object to be known (Foucault 2003, pp. 347–358).

This objection can hardly stand, however, if only because in contrast to Agamben, Foucault never posited the articulation between the empirical and the transcendental as in any way originary: the empirico-transcendental doublet belonged to the modern episteme that emerged in the nineteenth century and, at the time of writing, was already on the verge of dissolution as a result of innovations in the human sciences (Foucault 2002, pp. 407–420). This conjuncture between the empirical and the transcendental was result of a contingent mutation in the historical a priori of the knowledge of life, labour and language and would disappear in a similarly contingent manner, ‘like a face drawn in sand at the edge of the sea’ (Foucault 2002, p. 422). The doublet in question is therefore a transient articulation that must presuppose the distinction that it temporarily blurs. In contrast, the blurring produced by Agamben’s indistinctions is defined from the outside as the original activity of sovereign power that makes it impossible to access whatever preceded the blur in question. This is why for Agamben we are all virtually *homines sacri*: if sovereignty has always already captured bare life, what else could we ever be? Agamben’s question *Where Are We Now?* can therefore have only one answer: in the same place as always—the same state of exception, the same ban, the same camp that the originary activity of sovereign power has always already placed us in.

In contrast, our critical reconstruction of Agamben’s theory affirms that any articulation of sovereign power and bare life is not merely contingent but barely conceivable, as would be any other form of relation between them. While this conclusion clearly goes against the basic tenet of Agamben’s political theory, it also resonates with the more admittedly less developed and somewhat esoteric theme of non-relation that first appears in *Homo Sacer* 1, where the solution to the lethal logic of sovereign power was to be found precisely in ‘thinking ontology and politics beyond every figure of relation’ (Agamben 1998, p. 55). This rather ambiguous injunction was concretized in *The Use of Bodies*, where, drawing on Giorgio Colli’s notion of contact, Agamben developed the idea of non-relation as a proximity of elements, in which neither one presupposes, founds or excludes the other. ‘Contact is not a point of tangency nor a *quid* or a substance in which two elements communicate: it is defined only by an absence of representation, only by a caesura’ (Agamben 2016, p. 272). The two terms simply expose the void in the place where their articulation used to be. In this image, life is no longer included as excluded in the state of exception, stripped of its form and exposed to the violence of the law without content, but dwells freely in its form, from which it remains indissociable: ‘what has been divided from itself and captured in the exception now appears in its free and intact form’ (Agamben 2016, p. 273).

While the state of exception remains a paradigmatic form of relation, in which bare life is included into law as its negative foundation in the blurring of all distinctions between the empirical and the transcendental, a non-relational approach must on the contrary *draw distinctions* where they have been blurred by the logic of sovereignty. Life can appear ‘in its free and intact form’ only if there is a caesura between it and a variety of forms of power. Only when it is inaccessible to the inclusion into the apparatuses of power as a negative foundation, can the transcendental principle of bare life continue to generate myriad forms of life, none of which are reducible to what made them possible. Thinking ontology and politics beyond every figure of relation must therefore begin by dissolving the image of *homo sacer* and opening in its place a chasm that makes possible the continuous generation of forms of life that are *neither included into sovereign orders nor excluded from them*, but remain in non-relational contact with them.

Since these reflections were inspired by Agamben’s commentary on the coronavirus crisis, we may conclude them by addressing three implications of our reconstruction of (non) relation of sovereign power and bare life for the analysis of the pandemic. Firstly, as the state of exception loses its quasi-transcendental aura, it is no longer necessary to relate every instance of its promulgation to the darkest episodes of human history, be they conspiracies or genocides. Instead, it can be both analyzed and assessed as a specific instrument of governmental rationality, whose application may well be legitimate but is never necessary (cf. Foucault 2014, p. 79), which makes its criticism a permanently open possibility. Without the shadow of *homo sacer* hanging over us, it should be possible to assess the exceptional measures undertaken by governments worldwide in response to the pandemic without reducing them a priori to the fictive declarations of the Schmittian sovereign. Secondly, as the notion of bare life is no longer applicable to specific forms of life that one might find unappealing, we might refrain from moralizing invectives that deny some forms of life their very form and instead focus on the ways in which the pandemic does not merely demand the protection of our habitual forms of life but also serves as an impetus for their transformation in a more equitable and sustainable direction. Finally, as sovereign power and bare life are no longer related to one another, we might become more appreciative of the ongoing innovations and transformations of forms of life developed in response to the pandemic that are not blindly following any sovereign command, but rather arise from the imperatives of solidarity and mutual assistance.
